# The cardioprotective effect of whey protein against thioacetamide-induced toxicity through its antioxidant, anti-inflammatory, and anti-apoptotic effects in male albino rats

**DOI:** 10.3389/fvets.2025.1590722

**Published:** 2025-05-19

**Authors:** Zakiah Nasser Almohawes, Hanan A. Okail, Wafa A. Al-Megrin, Manal F. El-Khadragy, Mona A. Ibrahim, Ayah S. Fathalla, Doaa Soliman, Sherif R. Mohamed

**Affiliations:** ^1^Department of Biology, College of Science, Princess Nourah Bint Abdulrahman University, Riyadh, Saudi Arabia; ^2^Department of Zoology, Faculty of Science, Sohag University, Sohag, Egypt; ^3^Department of Zoology and Entomology, Faculty of Science, Helwan University, Cairo, Egypt

**Keywords:** thioacetamide, cardiotoxicity, whey proteins, histopathology, oxidative stress

## Abstract

**Introduction:**

Thioacetamide (TAA) is widely used as an experimental drug in liver disease studies and has been shown to exert toxicity across multiple organs. It has been linked to oxidative stress, inflammation, apoptosis, fibrosis, and epigenetic modifications. Whey protein (WP) provides an abundant supply of essential and non-essential amino acids that are vital for the human body. It is highly valued for its nutritional and biological properties, benefiting the immune, digestive, cardiovascular, neurological, and endocrine systems. This research sought to evaluate the possible protective effects of WP against TAA-induced cardiotoxicity in rats, emphasizing its antioxidant, anti-inflammatory, and anti-apoptotic mechanisms.

**Methods:**

A total of forty male rats were randomly divided into four groups, with each group containing ten rats: the control group, the TAA-treated group (100 mg/kg body weight), the WP-treated group (300 mg/kg body weight), and the WP + TAA group. The treatments were administered for three consecutive weeks.

**Results:**

The findings revealed that TAA exposure significantly reduced cardiac tissue activities of glutathione, superoxide dismutase, and catalase while markedly increasing malondialdehyde and nitric oxide activities. Additionally, TAA administration led to a significant elevation in inflammatory markers (TNF-α and IL-1β) and apoptotic markers (Bax and Bcl-2), along with increased caspase-3 gene expression in heart tissue. Serum levels of lactate dehydrogenase were also notably higher in the TAA-intoxicated group, accompanied by significant histopathological alterations, increased collagen fiber deposition, and a pronounced immunopositive reaction for TGF-β1 and NF-κB in heart tissue. However, pre-treatment with WP significantly alleviated TAA-induced cardiotoxicity by reducing oxidative stress, inflammatory response, and apoptotic markers in cardiac tissue.

**Discussion:**

The results indicate that WP supplementation offers protective effects and mitigates the cardiotoxicity triggered by TAA.

## 1 Introduction

Thioacetamide (TAA) is a sulfurous chemical substance that provides sulfide ions in the production of compounds that are both inorganic and organic (1). TAA has found extensive application in the manufacturing and production of rubber auxiliaries, electroplating additives, stabilizers, insecticides, crosslinking agents, catalysts, polymerization inhibitors, and photographic agents (2). TAA has important applications in drug manufacturing, where it is a key component in anticoagulant drugs (3). TAA can get inside the human body by a variety of means, including skin contact, ingestion of toxic vapors, inhalation, and polluted water and beverages (4). After being fed to rats, TAA rapidly converted into two metabolic intermediates: acetamide and TAA-S-oxide (5). TAA-S-oxide binds to certain proteins within the cell, disrupting cell permeability and storing calcium as well as mitochondrial function, resulting in cellular damage (6). Although TAA is well recognized to be toxic to the liver, previous research has demonstrated that exposure to TAA can have a variety of detrimental effects on the digestive system, kidneys, heart, bones, bone marrow, and brain, depending on the dose, duration, and environmental factors (3). The main mechanism for the toxic action of TAA on tissues is the increase of reactive oxygen species (ROS) generation (1, 3). Increased ROS levels lead to increased membrane lipid peroxidation, breakdown of antioxidant defenses, and the generation of inflammatory and apoptotic agents (7). Consequently, the excessive generation of ROS by TAA precipitates mitochondrial damage and DNA mutations, leading to apoptosis (8, 9) and fibrosis (10).

Cardiotoxicity is the incidence of cardiac malfunction caused by electrical or muscular injury (11). The primary cardiovascular menace factors that lead to cardiomyopathy, cardiac failure, myocarditis, and arrhythmia increase ROS generation, resulting in oxidative stress (12). In addition, cardiac tissue is more vulnerable to damage from ROS. This is due to its higher rate of oxidative metabolism and poorer antioxidant defense (13). Oxidative stress is one of the primary mechanisms that damages cells directly by interfering with mitochondrial activity and heart muscle energy generation. ATP depletion causes necrosis or apoptosis, which results in cell death (14).

ROS leads to mitochondrial dysfunction, fibrosis, and direct damage to cardiomyocytes, all contributing to heart failure (15). This may be explained by the link between mitochondrial function and oxidative stress, which reduces the effectiveness of the electron transport system in mitochondria and increases the production of superoxide radicals. These free radicals can further harm mitochondrial lipids, proteins, and DNA, potentially impairing mitochondrial function and decreasing ATP production, a vital element for cardiac muscle contraction (16). Also, inflammation, DNA damage, and abnormal cell communication are additional causes of cardiotoxicity (17). Previously, the short-term injection of TAA produced less cardiac damage in experimental animals due to the heart’s limited exposure to toxic substances (18). According to recent studies, prolonged exposure to higher doses of TAA in experimental animals can cause heart injury through ROS production and antioxidant system disturbance (1, 19).

Whey protein (WP), one of the most important elements of milk proteins, has become very popular due to its potential advantages in weight loss and muscle growth (20). WP is soluble proteins that make up around 20% of cow’s milk’s protein content. They are normally extracted from the liquid portion of milk during cheese manufacture, then filtered and dried (21). WP products vary in their level of processing and protein content, and they can be used as components in food products as WP isolate, which contains 90–95% protein and minimal amounts of minerals, lactose, and fat; WP concentrate, which contains 20–85% protein with various levels of minerals, lactose, and fat; or as WP hydrolysates, which hydrolyze to increase absorption and reduce antigenic reactions (22). In addition, WP properties can change depending on the casein precipitation technique, storage, heating, and other factors (23). The primary proteins in WP are immunoglobulins, glycomacropeptides, β-lactalbumin, β-lactoglobulin, and minor proteins, each of which has a variety of advantageous characteristics (20), and it can aid in the nutritional management of chronic diseases.

WP is often used by athletes who want to increase their body composition, muscle mass, or strength (24). Athletes require whey protein because it is rich in branched-chain amino acids, which are rapidly absorbed by muscles and utilized during exercise and strength training (25). It has been demonstrated that WP dramatically increases the synthesis of muscle proteins when taken either right before or right after an exercise course (26). In females, it appears that supplementing with whey protein improves muscle performance indicators (27), muscle protein synthesis, or the extent of muscle injury (28). It also appears to decrease the incidence of breast tumors and the multiplicity of breast tumors (29). Furthermore, WP has exceptional biological characteristics that benefit the immune, digestive, cardiovascular, neurological, and endocrine systems. Therefore, studies on their use to improve health through the creation of medications and the optimization of food product compositions are quite pertinent (30). Additionally, WP has been shown to be helpful in improving muscular mass and strength while also reducing weight and fatty tissue in patients with heart failure (31).

The mature heart is a “metabolic omnivore,” generating adenosine triphosphate (ATP) from a wide variety of food sources, including glucose, ketone bodies, fatty acids, and amino acids (32). Furthermore, improving cardiac function and energy generation in heart failure may be achieved by combining metabolic therapy with optimal nutrition (33).

According to previous literature, WP has a high nutritional and biological value, making it a great candidate for dietary and therapeutic nutrition in this study. Consequently, the present study sought to assess WP’s antioxidant potential to protect the heart from the damaging effects of TAA by leveraging its antioxidative, anti-inflammatory, and apoptosis-preventing properties. This is in addition to the few studies that have been conducted to determine TAA-induced heart injury.

## 2 Materials and methods

### 2.1 Chemicals

Thioacetamide (TAA) was procured from Sigma-Aldrich Corp., St. Louis, MO, USA, whereas whey protein (100% natural whey protein concentrate) was obtained from Imtenan Health Shop, Cairo, Egypt.

### 2.2 Experimental protocol

A total of forty adult male albino rats, aged 7–9 weeks and weighing 180–200 g, was acquired from the animal facility at Sohag University, Egypt. The rats underwent a one-week acclimatization period in laboratory conditions with unrestricted food and water access. The study was conducted by the guidelines established by the internal research regulatory board and the animal ethics committee at Sohag University, Egypt (Faculty of Science: Department of Zoology), under approval number (CSRE-46-24).

Group I (Control, *n* = 10): Rats in this group received distilled water orally along with intraperitoneal injections of normal saline (NaCl 0.9%) 5 days per week for 3 weeks.

Group II (TAA, *n* = 10): This group was administered TAA dissolved in NaCl 0.9% intraperitoneally at a dosage of 100 mg/kg/day, 5 days per week for 3 weeks (34).

Group III (WP, *n* = 10): Rats received an oral gavage of whey protein (WP) at 300 mg/kg per day (35) for 3 weeks.

Group IV (WP + TAA, *n* = 10): These rats were given 300 mg/kg WP orally, which was administrated 2 h before TAA administration (100 mg/kg, 5 days per week) for 3 weeks.

After the experiment, the rats underwent euthanasia through an intraperitoneal injection of thiopental sodium at a dose of 20 mg/kg, and blood samples were collected in anticoagulant-free tubes. The blood was maintained at room temperature for 30 mins before undergoing centrifugation at three thousand five hundred rpm to isolate the serum. The hearts were dissected and cleaned, with one portion preserved for histological analysis; meanwhile, the other was weighed and homogenized in a chilled medium. The homogenate was centrifuged at 1,500 × g for 10 min at 4°C, and the supernatant was collected for biochemical analysis.

### 2.3 Biochemical assays

The biochemical parameters were assessed in serum (for LDH) and in homogenized heart tissue (for oxidative and antioxidant as well as inflammatory and apoptotic markers) following the instructions of the respective kit manufacturers.

#### 2.3.1 Measurement of lactate dehydrogenase (LDH)

LDH, a cardiac biomarker, was measured colorimetrically in serum based on the method outlined by Cabaud et al. (36). Briefly, Serum LDH activity was measured colorimetrically by monitoring NADH production at 340 nm during lactate-to-pyruvate conversion. Samples were incubated with lactate substrate and NAD + cofactor in phosphate buffer (pH 7.5, 37°C) for 30 min. Results were calculated against a standard curve (0–500 U/L) with quality controls ensuring <5% CV.

#### 2.3.2 Analysis of oxidative and antioxidant markers

All biochemical evaluations were carried out as per the manufacturer’s protocols (Diagnostic, Giza, Egypt). The TBARS assay was utilized to measure the malondialdehyde (MDA) level for oxidative stress assessment (37). Nitric oxide (NO) concentration was determined following Archer (38), where it converts to nitrous acid and reacts with sulfanilamide and N-(1-naphthyl) ethylenediamine, with absorbance measured at five hundred forty nanometers. Glutathione (GSH) activity was determined using the approach described by Beutler et al. (39), involving the reduction of 2-nitrobenzoic acid, yielding a yellow chromogen measurable at 405 nm. Superoxide dismutase (SOD) activity was determined following Nishikimi et al. (40) by measuring its capacity to suppress nitroblue tetrazolium dye reduction which is brought on by phenazine methosulphate. Catalase (CAT) activity was determined as per Aebi (41), where H₂O₂ degradation is halted using a catalase inhibitor, and the residual H₂O₂ reacts with 4-aminophenazone, 3,5-dichloro-2-hydroxybenzene sulfonic acid, and peroxidase to form a chromophore.

#### 2.3.3 Evaluation of inflammatory and apoptotic markers

Cardiac levels of interleukin-1 beta (IL-1β), tumor necrosis factor-alpha (TNF-α), Bcl-2, and Bax were quantified in heart tissue homogenates using commercial sandwich ELISA kits (Sunlong Biotec, China), according to the manufacturer’s instructions. Tissues were homogenized, centrifuged at 1,500 × g for 10 min at 4°C, and the supernatants were collected. Total protein concentration was determined (Bradford or BCA assay) for normalization. All samples, standards, and controls were assayed in triplicate. Standard curves were generated using serial dilutions of recombinant proteins over the following ranges: IL-1β (15.6–1,000 pg./mL), TNF-α (7.8–500 pg./mL), Bcl-2 (31.2–2000 pg./mL), and Bax (62.5–4,000 pg./mL), with curve fitting via 4-parameter logistic regression (R^2^ > 0.99). Assay validation showed intra-assay CVs < 8%, inter-assay CVs < 12%, and spike recovery between 85–115%. Sensitivities were: IL-1β (4.7 pg./mL), TNF-α (2.3 pg./mL), Bcl-2 (9.5 pg./mL), and Bax (18.6 pg./mL). Absorbance was measured at 450 nm (reference at 570 nm), and concentrations were interpolated from standard curves and normalized to total protein when applicable. Full validation data and standard curves are presented in Supplementary Figure S1 and Supplementary Table S1.

### 2.4 Real-time qPCR analysis

Total RNA was extracted from 50 mg of left ventricular heart tissue homogenized in TRIzol® (Invitrogen), using the High Pure RNA Isolation Kit (iNtRON) with on-column DNase I treatment. RNA purity (A260/A280 = 1.9–2.1) and integrity (RIN > 7.0) were confirmed using NanoDrop™ and Agilent Bioanalyzer, respectively. cDNA was synthesized from 1 μg of total RNA using the RevertAid First Strand cDNA Synthesis Kit (Thermo Scientific) with oligo(dT)18 primers; no-reverse-transcription controls were included. qPCR was performed using FastStart Universal SYBR Green Master Mix (Roche) with validated primers (Table 1) spanning exon-exon junctions. Reactions (20 μL) were run in technical triplicates under the following conditions: 95°C for 10 min, followed by 40 cycles of 95°C for 15 s and 60°C for 1 min, and a melt curve (65–95°C). Specificity was confirmed via melt curve (single peak) and 2% agarose gel electrophoresis. Primer efficiency (90–110%) was calculated from a 5-point dilution series (R^2^ > 0.99). Relative gene expression was normalized to GAPDH using the 2^−ΔΔCt method (42), with stable reference gene expression confirmed by geNorm (M-value < 0.5). All procedures adhered to MIQE guidelines.

**Table 1:** Forward and reverse primer sequences used in qPCR.

Gene	Forward Primer (/5 ------ /3)	Reverse Primer (/5 ------ /3)
Caspase3	GGTATTGAGACAGACAGTG	CATGGGATCTGTTTCTTTGC
GAPDH	CAACTCCCTCAAGATTGTCAGCAA	GGCATGGACTGTGGTCATGA

### 2.5 Histological and histochemical analysis

The histological analysis was performed on left ventricular tissue sections to assess myocardial structure and fibrosis. For each heart, three transverse sections (5-μm-thick) were taken from the mid-ventricular region to ensure consistency. The sections were deparaffinized, rehydrated, and stained with hematoxylin and eosin (H&E) following standard protocols (43): hematoxylin (nuclear stain, 5–10 min) differentiated in acid alcohol, blued in Scott’s solution, and counterstained with eosin (cytoplasmic stain, 1–2 min). H&E-stained sections were used to evaluate myocyte nuclear morphology and inflammatory infiltrates under light microscopy. For fibrosis assessment, parallel sections underwent Masson’s trichrome staining: Weigert’s hematoxylin (5 min) for nuclei, Biebrich scarlet-acid fuchsin (5 min) for cytoplasm, phosphomolybdic acid differentiation (5 min), and aniline blue (5 min) to highlight collagen (blue) versus muscle (red) (44).

### 2.6 Immunohistochemical staining

Cardiac tissue sections were processed for immunohistochemistry (IHC) using rabbit monoclonal anti-NF-κB p65 (Abcam ab16502, Cambridge, UK) and rabbit monoclonal anti-TGF-β1 (Abcam ab215715, Cambridge, UK) antibodies following the manufacturer’s standardized protocols. Cardiac tissues were fixed in 10% neutral buffered formalin (24–48 h, RT), then dehydrated in graded ethanol (50–100%, 1 h each), cleared in xylene (2×1 h), and embedded in paraffin (56–58°C). Sections (4-5 μm) were cut using a microtome, floated on 40°C water, mounted on poly-L-lysine slides, and dried (37°C, overnight). For staining, slides were deparaffinized in xylene (2×10 min), rehydrated through ethanol (100–50%, 5 min each), and rinsed in distilled water. For antigen retrieval, slides underwent heat-mediated epitope unmasking in citrate (pH 6.0) or Tris-EDTA (pH 9.0) buffer (95°C, 15–20 min), cooled 30 min, then washed in PBS (3×5 min). Endogenous peroxidase was blocked with 3% H₂O₂/methanol (10 min, RT), followed by 5% goat serum blocking (1 h, RT). Primary antibodies (NF-κB p65 1:100–500; TGF-β1 1:50–200 in 1% BSA/PBS) were incubated overnight at 4°C. Detection used Abcam’s HRP/DAB kit (ab80436) with secondary antibody (1 h, RT). DAB development (1–10 min) was monitored microscopically before water termination. Sections were then dehydrated (50–100% ethanol), cleared in xylene, and DPX-mounted. Controls included antibody omission and positive tissue samples. Analysis used ImageJ software.

### 2.7 Morphometric analysis

Cardiac tissue lesions including vacuolar degeneration, loss of cross striation, coagulative necrosis, and fibrosis of interstitial tissue were classified as no, mild, moderate, or severe changes using a percentage (0–3) scale (45). Ten randomly selected high-power fields (400× magnification) per heart tissue section were analyzed to determine the mean area percentage of collagen fibers (Fibrosis measuring blue collagen-positive areas as a percentage of total tissue area), TGF-β1-positive regions, and NF-κB-positive reactions. Area percentage was quantified using image analysis software (ImageJ, version 1.46, NIH, USA). To minimize bias, blinded analysis was performed by two independent observers, with results averaged. This standardized approach ensured robust assessment of pathological changes.

### 2.8 Statistical analysis

Data processing and statistical evaluation were conducted using GraphPad Prism 5 (GraphPad Inc., CA, USA). Experimental results are reported as means ± SD, reflecting data distribution characteristics. Group comparisons employed one-way ANOVA to assess overall significance, with Tukey’s HSD post-test applied when ANOVA indicated significant differences (*p* < 0.05), enabling specific group comparisons while controlling for multiple testing errors.

## 3 Results

### 3.1 Lactate dehydrogenase (LDH)

**Figure 1:**
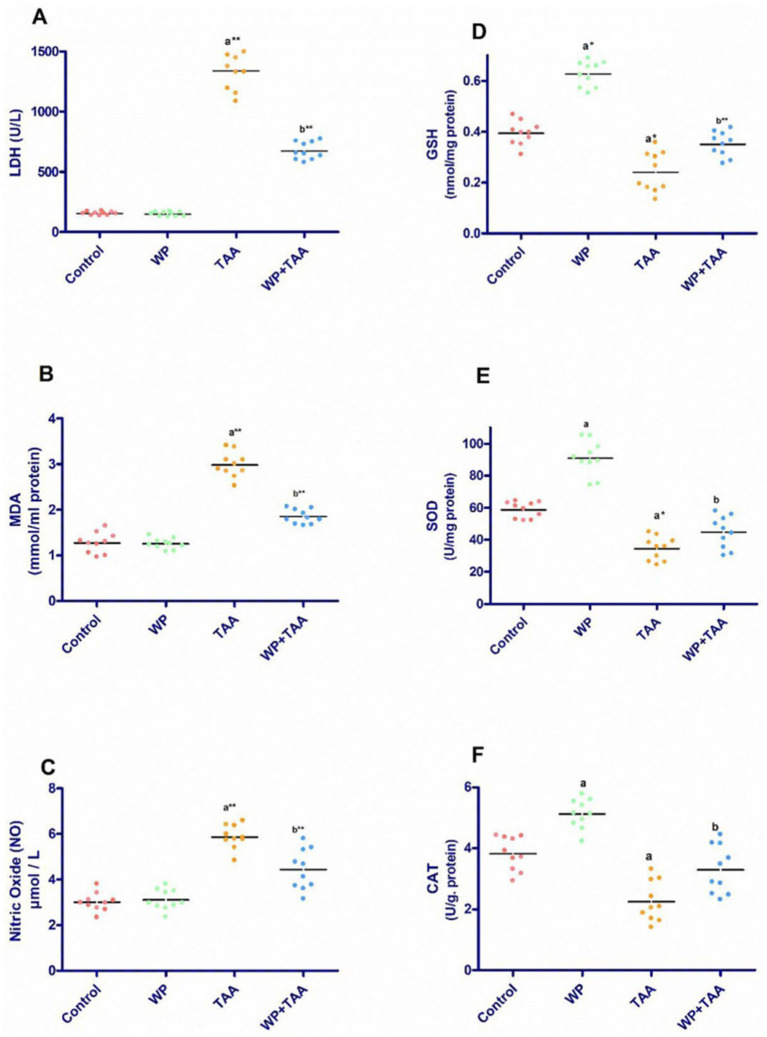
Effect of whey protein (WP) administration on the cardiac levels of **(A)** Lactate dehydrogenase (LDH), **(B)** Malondialdehyde (MDA), **(C)** Nitric Oxide (NO), **(D)** Glutathione (GSH), **(E)** superoxide dismutase (SOD) and **(F)** catalase (CAT) of rats treated with thioacetamide (TAA). Significance: *p* ≤ 0.05 (a) relative to the control group, (b) relative to the TAA group, (*): *p* ≤ 0.01, (**): *p* ≤ 0.001.

Serum LDH concentrations across all experimental groups are depicted in Figure 1A. Administration of TAA led to a significant (*p* ≤ 0.001) elevation in serum LDH levels relative to the untreated control group. However, pre-treatment with WP in TAA-exposed rats significantly improved LDH levels (*p* ≤ 0.001) in contrast to those receiving TAA alone.

### 3.2 Oxidative stress indicators

Figures 1B,C illustrates the cardiac homogenate level of malondialdehyde (MDA) along with nitric oxide (NO) across different groups. Exposure to TAA resulted in a notable (*p* ≤ 0.001) increase in both MDA and NO level when assessed against the control rats. However, concurrent administration of WP with TAA significantly lowered MDA (*p* ≤ 0.001) together with NO (*p* ≤ 0.01) activity when juxtaposed with rats subjected to TAA alone.

### 3.3 Antioxidant defence

Rats treated with TAA displayed a considerable (*p* < 0.01) reduction in cardiac glutathione (GSH) level relative to controls, reflecting oxidative stress in cardiac tissue. Nevertheless, WP supplementation in TAA-exposed rats significantly (*p* ≤ 0.001) restored GSH activity (Figure 1D). Similarly, TAA administration significantly (*p* ≤ 0.05) diminished superoxide dismutase (SOD) activity alongside catalase (CAT) activity in cardiac tissue. Conversely, WP supplementation in TAA-treated rats significantly (*p* ≤ 0.05) enhanced SOD activity in addition to CAT activity (Figures 1E,F).

### 3.4 Inflammatory and apoptotic markers

**Figure 2:**
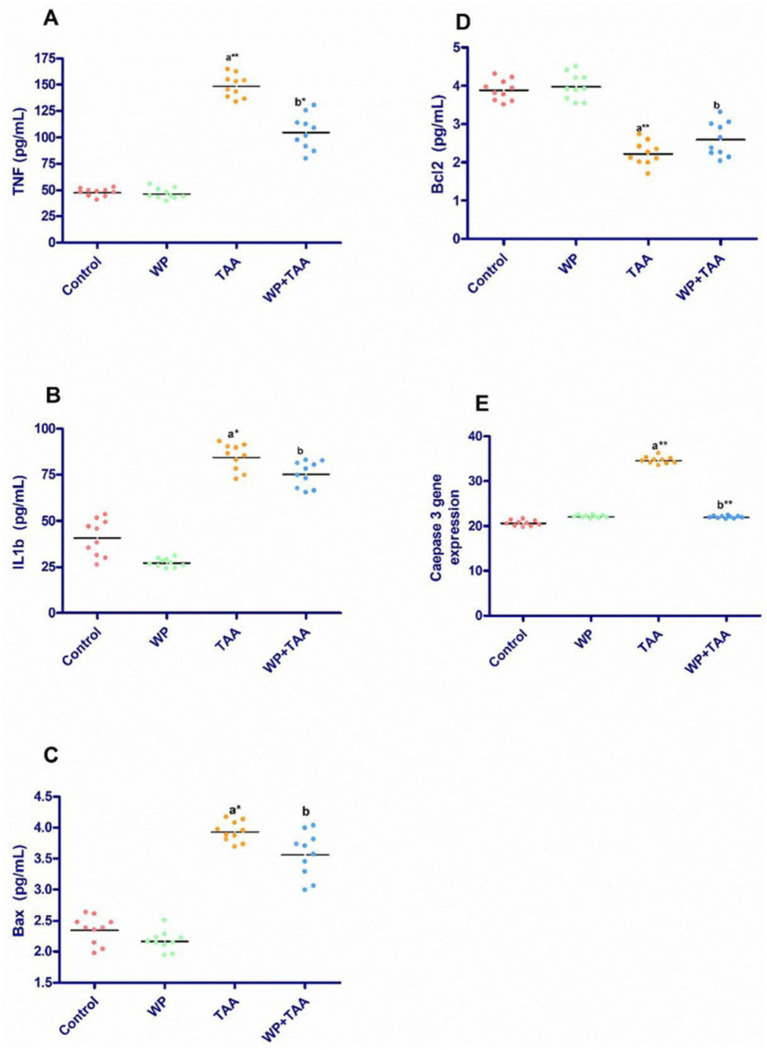
Effect of whey protein (WP) administration on the cardiac levels of **(A)** tumor necrosis factor (TNF-a), **(B)** interleukin-1 beta (IL-1ß), **(C)** Bcl-2–associated X protein (Bax), **(D)** B-cell lymphoma protein 2 (Bcl-2) and **(E)** Caspase 3 gene expression of rats treated with thioacetamide (TAA). Significance: *p* ≤ 0.05 (a) relative to the control group, (b) relative to the TAA group, (*): *p* ≤ 0.01, (**): *p* ≤ 0.001.

Figure 2 presents the cardiac tissue levels of IL-B1, TNF-*α*, Bax, and Bcl-2. Rats receiving TAA exhibited a marked (*p* ≤ 0.001) increase in TNF-α coupled with a substantial (*p* ≤ 0.001) rise in IL-B1 levels about the control group. However, WP supplementation significantly (*p* ≤ 0.05) reduced both IL-B1 and TNF-α concentrations in TAA-exposed rats when weighed against the TAA-only group (Figures 2A,B). Additionally, Bax levels were significantly (*p* < 0.01) elevated, whereas Bcl-2 levels were notably (*p* ≤ 0.001) reduced in the TAA group relative to controls. In contrast, co-administration of WP with TAA led to a significant (*p* ≤ 0.05) decrease in Bax levels together with a notable (*p* ≤ 0.05) increase in Bcl-2 levels when evaluated against the TAA-treated group (Figures 2C,D).

### 3.5 Real-time qPCR analysis

As shown in Figure 2E, caspase-3 gene expression in cardiac tissue was significantly (*p* ≤ 0.001) upregulated in TAA-treated rats about the untreated control group. However, WP supplementation in TAA-exposed rats led to a considerable (*p* ≤ 0.001) reduction in cardiac caspase-3 expression when set against the TAA-only group. Moreover, when WP was administered alongside TAA, caspase-3 expression remained within normal ranges, similar to the control group.

### 3.6 Histological investigations

**Figure 3:**
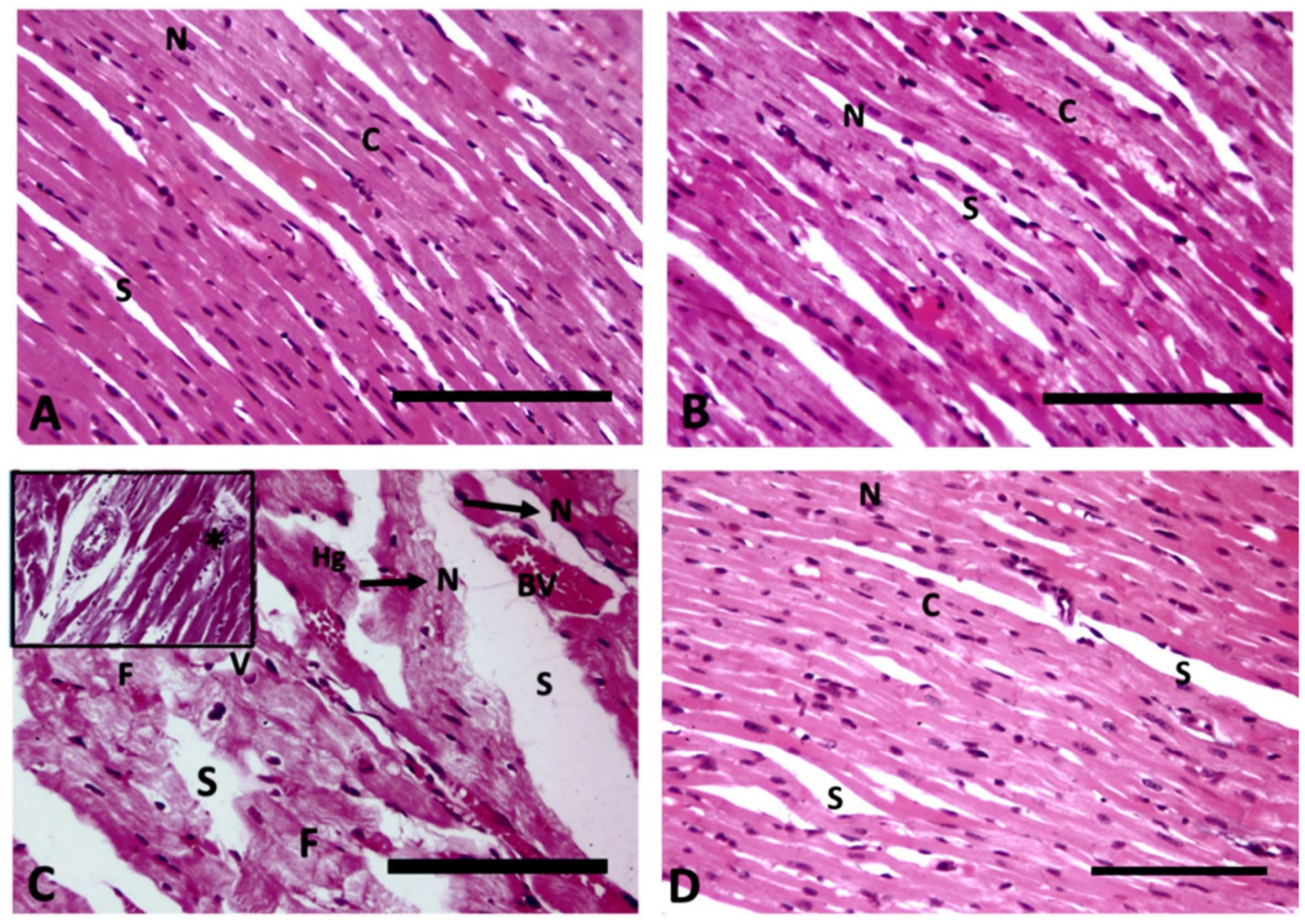
Longitudinal section of heart muscle (400× magnification); **(A)** Control rats exhibit typical branching striated cardiac myocytes with intact acidophilic cytoplasm (C), centrally positioned nuclei (N), and interfibrous spaces (S). **(B)** Rats receiving WP have normal cardiac myocyte histology, which includes intact acidophilic cytoplasm (C), centrally placed nuclei (N), and interfibrous gaps (S). **(C)** TAA-treated rats exhibit myocyte disorganization with cytoplasmic vacuolization (V), pyknotic nuclei (N) separated by big spaces (S), myocyte fragmentation (F), haemorrhage (Hg), dilated congested blood vessels (BV) and inflammatory infiltrate (star) in the upper insert. **(D)** Rats treated with WP and TAA have approximately normal cardiac myofiber architecture, with intact acidophilic cytoplasm (C), centrally-located nuclei (N), and separated by fairly wide interfibrous gaps (S), as well as some pyknotic nuclei (arrow). (H&E, Bar = 100 μm).

The longitudinal section of heart muscle stained with hematoxylin and eosin of control rats exhibits typical branching striated cardiac myocytes with intact acidophilic cytoplasm, centrally positioned nuclei, and interfibrous spaces (Figure 3A). Rats receiving WP have normal cardiac myocyte histology, which includes intact acidophilic cytoplasm, centrally placed nuclei, and interfibrous gaps (Figure 3B). The cardiac architecture in TAA-treated rats exhibited significant histological alterations, including myocyte disorganization with cytoplasmic vacuolization; pyknotic nuclei separated by big spaces; myocyte fragmentation; hemorrhage; and dilated, congested blood vessels (Figure 3C). Treatment of rats with WP plus TAA has approximately normal cardiac myofiber architecture, with intact acidophilic cytoplasm, centrally located nuclei separated by fairly wide interfibrous gaps, and some pyknotic nuclei. The architecture of the heart tissue remained intact, and there were fewer areas of degeneration than with TAA alone (Figure 3D).

**Figure 4:**
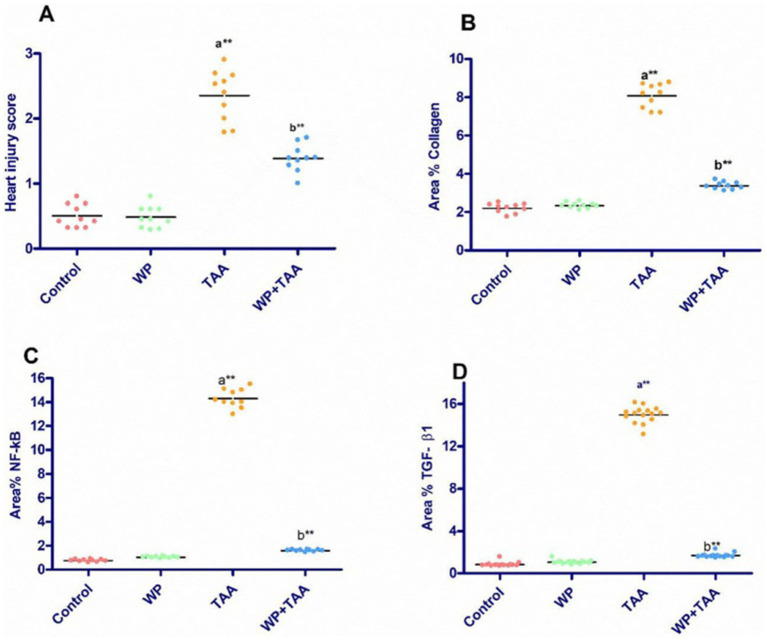
The heart injury score and mean area percent of collagen fiber, nuclear factor-kappa B (NF-κB), transforming growth factor β (TGF-β1) positive reactions in heart tissue. Data presented as mean ± SEM of ten animals per group. Significance: *p* ≤ 0.05 **(A)** relative to the control group, **(B)** relative to the TAA group, (*): *p* ≤ 0.01, (**): *p* ≤ 0.001.

The scoring of heart injury in the sections revealed significant (*p*< 0.001) damage in the rats receiving TAA in the form vacuolar degeneration of myocytes, loss of cross striation, coagulative necrosis, and fibrosis of interstitial tissue in heart tissues compared to the control group. On the other hand, the treatment of WP with TAA prevented heart injury and resulted in a substantial (*p*< 0.001) reduction in heart injury score compared to the rats treated with TAA (Figure 4A).

### 3.7 Histochemical results

**Figure 5:**
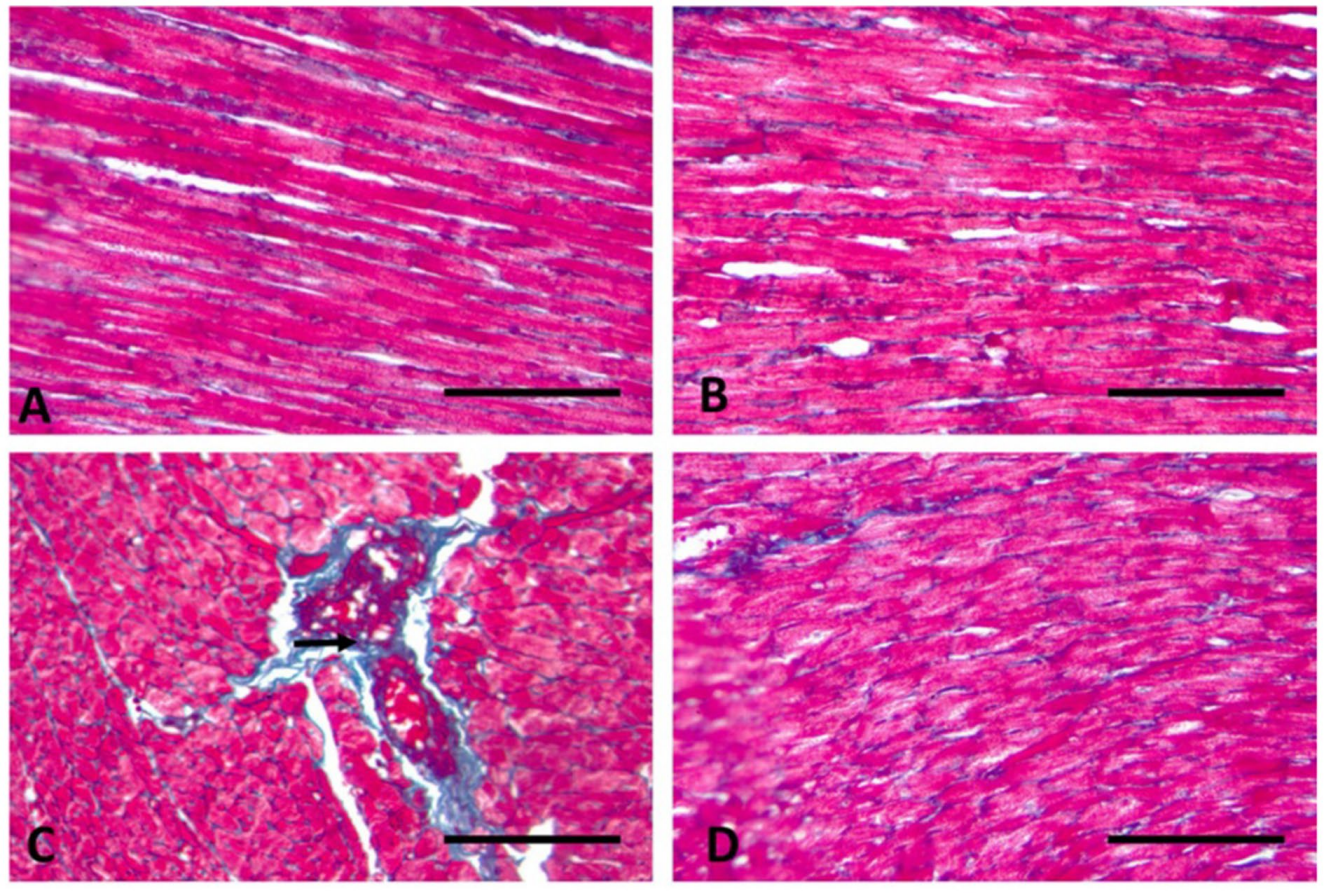
Longitudinal section of the heart muscle (400× magnification). **(A)** Control rats have normal sparse blue-stained collagen fibers that are evenly distributed in the endomysium (arrows). **(B)** In rats given WP, normal collagen fibers are mildly dispersed in the endomysium. **(C)** TAA-treated rats display significant collagen accumulation in the endomysium and around dilated, congested blood vessels (arrow). **(D)** Throughout rats treated with WP and TAA, cardiac collagen fibers are mild to moderately disperse throughout the endomysium. (Masson trichrome; Bar = 100 μm).

Longitudinal sections of the heart muscle stained with Masson trichrome in control rats have normal, sparse, blue-stained collagen fibers that are evenly distributed in the endomysium (Figure 5A). TAA-treated rats display significant collagen accumulation in the endomysium and around dilated, congested blood vessels (Figure 5C). In rats given WP, normal collagen fibers are mildly dispersed in the endomysium (Figure 5B). While rats are treated with WP and TAA, cardiac collagen fibers are mildly to moderately disperse throughout the endomysium (Figure 5D). The area % of collagen fibers was significantly higher (*p* ≤ 0.001) than in the control group. WP-treated rats showed a considerably (*p* ≤ 0.001) lower collagen fiber area percent than the THA-treated group (Figure 4B).

### 3.8 Immunohistochemical results

**Figure 6:**
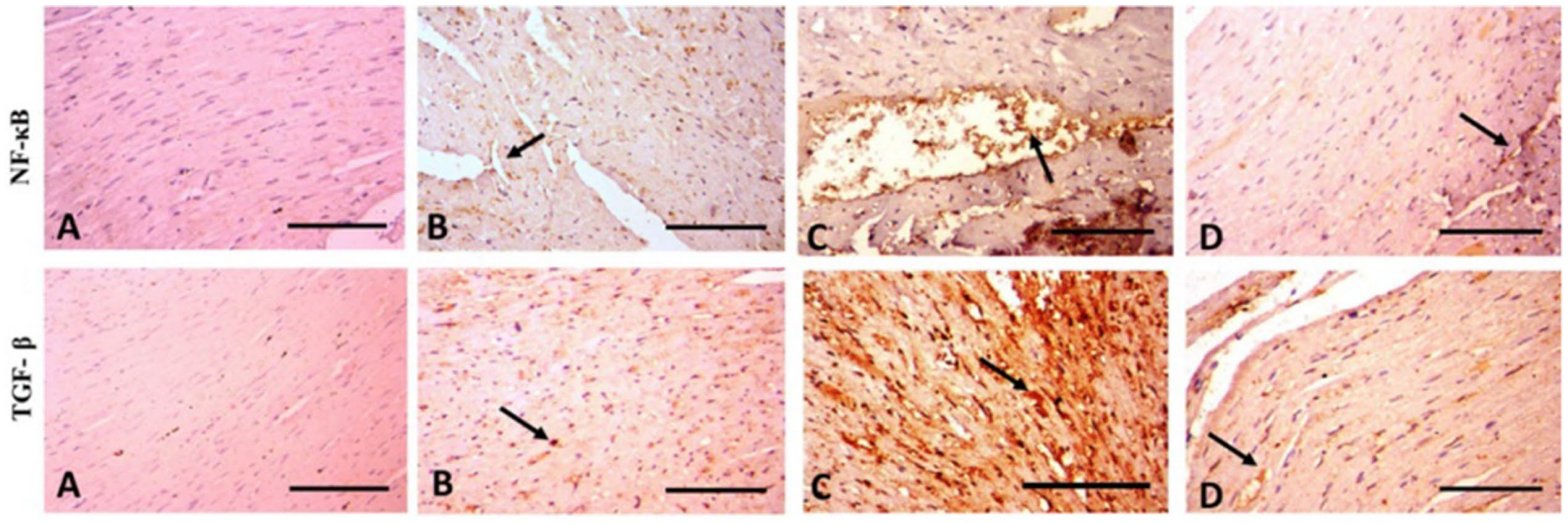
Photomicrograph of rat heart of anti-NF-κB and anti-TGF-β1 (400× magnification). **(A)** Control rats have a normal sparse immunopositive reaction. **(B)** In rats given WP normal spare immunopositive reactions (arrow) appear. **(C)** TAA-treated rats display severe immunopositive reaction around blood vessels (arrow). **(D)** In rats treated with WP and TAA, cardiac fibers appear with mild immunopositive reaction (arrow). Scale Bar = 100 μm.

According to the immunohistochemical analysis, both NF-κB and TGF-β1 marker expression were not detectable in the control group’s cardiac tissue (Figure 6A). The WP group’s cardiac tissue exhibited low expression of NF-κB and TGF-β1 markers (Figure 6A). TGF-β1 and NF-κB expression levels in the cardiac tissue of TAA-treated animals around blood vessels were higher than those of the rats in the control group (Figure 6C). Rats treated with WP and TAA had lower NF-κB and TGF-β1 expressions in heart tissue, and cardiac fibers had milder immunopositive reactions compared to rats treated with TAA (Figure 6D). The area percent of TGF-β1 and NF-κB expressions was significantly higher (*p* ≤ 0.001) than in the control group. The NF-κB and TGF-β1 expression area percent was significantly (*p* ≤ 0.001) lower in WP + TAA-treated rats than in the TAA-treated group (Figures 4C,D respectively).

## 4 Discussion

The toxic effect of TAA on heart tissue is based on the commencement of ROS generation, which induces other factors like inflammatory and apoptotic agents (45). TAA-induced ROS production has numerous detrimental effects on heart cells, leading to eventual destruction of cardiac tissue (46). WP contains several bioactive nutrients that may help stop tissue damage brought on by oxidative stress through the enhancement of antioxidant components. Our investigation shows that WP’s anti-inflammatory, anti-apoptotic, and antioxidant qualities provide a cardioprotective benefit against TAA-induced cardiotoxicity.

In this study, TAA exposure significantly increased MAD and NO levels and declined GSH, SOD, and CAT activities in heart tissue homogenate. These results align with earlier research on TAA-induced cardiotoxicity in rats due to elevated ROS and a disturbance in the antioxidant balance (1, 19). Also, El-Gendy et al. (45) found that TAA injection induced cardiac damage and caused a substantial decrease in total antioxidant capacity besides a significant increase in MDA, which is one of the byproducts of lipid peroxidation, indicating a rise in damage to the membrane (47). NO is a transient lipophilic molecule that is linked to the production of many reactive intermediates (48). Regarding gene expression profile studies, Michalski et al. (49) reported that TAA disrupts the antioxidant response element. As a result, this causes a reduction in the expression of genes linked to antioxidant function.

Additionally, TAA dramatically increased MDA while decreasing GSH and SOD activities in liver tissue of mice (50). Moreover, Jorgačević et al. (51) stated that the decreased CAT and GSH in animals treated with TAA may be related to the TAA derivative combination, which nullifies the antioxidant system and can cause the accumulation of H₂O₂. According to Han et al. (52), commonly regulated genes in the liver and kidney from TAA-administered animals were linked to xenobiotic metabolism, lipid metabolism, and oxidative stress.

Both *in vivo* and *in vitro* studies demonstrated that increasing antioxidant capacities (GSH, SOD, and CAT) in cells through nutrition can enhance longevity and well-being (53). According to our findings, the WP-treated group exhibited an improved antioxidant capacity in heart tissue, evidenced by higher GSH levels and increased CAT and SOD activity. This outcome is expected since whey proteins are rich in cysteine, a fundamental building block for GSH synthesis. These findings align with those of Bounous et al. (54), who reported that aged C7BL/6NIA male mice fed a diet high in WPC had significantly higher GSH levels in their livers and hearts compared to those fed a casein-rich or control chow diet for the same duration. Additionally, Falkowski et al. (55) demonstrated a significant increase in GSH and SOD levels in the salivary glands of rats fed WPC-80 for 14 days compared to control rats. Furthermore, WPC significantly increased the SOD and CAT activity in C2C12 muscle cells treated with 0.1 to 0.4 mg/mL of WP for 24 h, both in stressed cells exposed to 0.75 mM H2O2 for 1 h and in nonstressed cells, compared to those not treated with WP (56).

The present investigation further demonstrated that TAA treatment caused cardiotoxicity by significantly increasing LDH activity in heart tissue homogenate and causing severe histopathological alterations in cardiac tissue. The current findings are consistent with a study that observed histopathological changes, including cardiac tissue bleeding, necrosis, eosinophil infiltration, edema, and vacuolar degeneration in rats treated with 200 mg/kg of TAA for 3 weeks (1). Moreover, elevated ROS formation and MDA activities may be the cause of TAA-induced cardiac tissue damage, which damages cellular constituents and modifies cell structure and function (19). Similarly, Kundu et al. (57) linked the harmful effects of TAA to the poisonous organo-sulfur material of TAA, which is swiftly transformed to ROS. In a recent study, TAA treatment resulted in histopathological changes, including inflammatory cell infiltration, myocyte vacuolar degeneration, and perivascular and interstitial tissue (45). It has been reported that the heart cell includes CK-MB and LDH enzymes, which are produced in the bloodstream as a result of enhanced lipid peroxidation (58). Consequently, the present detectable histopathological changes in heart tissue, indicating damage and injury to the heart cells, may be the cause of the elevated cardiac enzyme activities. This recommendation aligns with the results observed by El-Gendy et al. (45).

In this study, prior administration of WP alongside TAA effectively lowered MDA as well as NO concentrations while enhancing GSH, SOD, and CAT activities. These results were accompanied by an improvement in the histological structure of heart tissue. It has been demonstrated that probiotic therapy significantly reduced heart failure and improved cardiomyocyte hypertrophy and decreased oxidative stress (59). However, there was an improvement in the levels of oxidative stress markers along with antioxidant enzymes in the heart tissue of rats consuming a high-fat diet after WP supplementation in combination with endurance exercise (60).

Thioacetamide (TAA) suppresses antioxidant gene expression and enzyme activity through multiple interconnected mechanisms. Upon hepatic metabolism by cytochrome P450 2E1 (CYP2E1), TAA generates reactive metabolites that produce excessive reactive oxygen species (ROS), leading to oxidative stress and glutathione (GSH) depletion (61). This oxidative burden impairs critical antioxidant enzymes, including glutathione peroxidase and superoxide dismutase. Furthermore, TAA disrupts the Nrf2-Keap1 signaling axis, preventing Nrf2 translocation to the nucleus and subsequent activation of antioxidant response element (ARE)-regulated genes such as heme oxygenase-1 and NAD(P)H quinone oxidoreductase 1 (62). Prolonged TAA exposure also triggers inflammatory mediators like NF-κB and TNF-*α*, which antagonize Nrf2 activity while promoting oxidative damage. Emerging evidence suggests that TAA may also silence antioxidant genes through epigenetic mechanisms, including DNA methylation and histone modifications (63, 64).

According to another study, consuming WP supplements helps the body meet its needs for minerals and amino acids that are necessary for maintaining the heart and preventing aging. Whereas, supplementation with WP improved the oxidative stress and antioxidant enzymes with a marked restoration in the heart histological structure and the activities of LDH, cTn1, and CK-MB enzymes in aged heart tissue in Wistar Albino rats (65). Furthermore, WP administration improved cardiomyopathy of either high-fat diet mother rats (66) or their offspring (67).

Moreover, several studies have shown comparable results in other tissues. In this regard, the dietary WP consumption showed signs of improvements in hepatorenal toxicity induced by cadmium (68), in hepatotoxicity of rats fed a diet high in both fat and fructose (69) and in myopathy of mice induced by sepsis (70).

A growing body of research indicates that the inflammatory response is linked to oxidative stress. Inflammation results from any change in the structural integrity of tissues, and it triggers several repair mechanisms to assist the tissue in returning to its normal state (71). Additionally, IL-1β, a member of the interleukin-1 class, causes resident immune cells to become activated and additional leukocytes to migrate to the wounded liver, causing chronic inflammation (72). The pro-inflammatory cytokine TNF-*α* is generated immediately by neutrophils in response to tissue injury. In this study, TAA-injected rats showed an elevation of cardiac IL-1β and TNF-α levels. These findings were in line with those of El-Gendy et al. (45), who found a strong correlation between elevated IL-1β and TNF-α immune expression and histological evidence of cardiac necrosis. TNF-α exacerbates cardiac failure by upsetting the homeostasis-preserving mechanism, causing dysfunction and inhibiting anti-inflammatory reactions. Additionally, IL-1β and TNF-α levels in hepatic tissue were significantly elevated after receiving TAA treatment (100 mg/kg) for 2 weeks (34). Similarly, TAA caused a significant elevation in the expression of p65, NF-κB, and IL-1β (50).

In the present outcomes, TAA and WP administration successfully decreased inflammatory markers, as shown by a decline in the elevated cardiac IL-1β along with TNF-α levels relative to the group treated with TAA. These findings aligned with those of Prokopidis (73), who found that WP and soy protein are effective dietary treatments for decreasing serum levels of IL-6 along TNF-α. Furthermore, WP had a greater ability to reduce inflammatory markers such as CC chemokine ligand-5 and monocyte chemotactic protein-1 after high-fat meals when taken at a dose of 45 g/day in middle-aged and overweight individuals (74). Also, Ebaid et al. (75) found oral supplementation with WP to a wounded diabetic group dramatically decreased NO along with MDA levels with a significant restoration in the GSH level as well as the levels of inflammatory markers TNF-α, IL-4, and IL-6, along with IL-1b, to the levels in controls. In addition, Ma et al. (76) reported that WP’s anti-inflammatory activities are most likely attributable to its many bioactive peptide contents. Similarly, 30-month-old rats exhibited a depletion of the increased inflammatory markers (NF-kB and TNF-α) when supplemented with WP (65).

In the present biochemical data of apoptotic markers, Bax and Bcl-2, TAA-treated rats showed a rise in cardiac Bax level along with a decline in Bcl-2 levels, which were confirmed by increased expression of the caspase 3 gene. Furthermore, TAA treatment raised the levels of p53 and hydrolyzed caspase-3 while decreasing Bcl-2 (77). Similarly, 8 weeks of treatment with TAA (200 mg/kg) caused a significant elevation in the gene expression of caspase-3 and Bax with a drop in Bcl-2 gene expression in mice (50). Likewise, Wang et al. (78) found that TAA-treated zebrafish larvae showed a rise in the expression of Bax and p53 with a decline in Bcl-2 expression. Recent studies demonstrated that TAA treatment raised the Bax gene expression while decreasing the caspase-8 gene expression (79) or decreased both Bcl-2 and the Bax/Bcl-2 ratio (45).

Conversely, TAA and WP administration successfully reduced apoptosis, as shown by a decline in the Bax level and an elevation of the Bcl-2 level, which were supported by decreased caspase 3 gene expression compared to rats treated with TAA. WP’s protective effect against oxidative stress could be attributed to its ability to maintain mitochondrial membrane integrity and modulate the mitochondrial apoptotic process. Several studies demonstrated that camel WP has anti-apoptotic properties and significantly restored the ratio of Bax/Bcl-2 in diabetic mice (80) as well as the elevated caspase-3 and caspase-9 in heat-stressed mice (72). Likewise, supplementation with WP improved caspase-3 of aged heart tissue in Wistar Albino rats (65). Recent research has shown that WP hydrolysate dramatically reduced Ca2+ influx and controlled the Bax/Bcl2 ratio in oxidative stress-induced neuronal cell death, indicating WP has neuroprotective properties by inhibiting apoptosis (81). In a rotenone-induced Parkinson’s disease rat model, WP was shown to have a neuroprotective effect by increasing GSH levels and lowering caspases 8 and 9, as well as cytochrome C (83).

Myocardial fibrosis is a common side effect of most cardiac pathologic diseases due to the accumulation of extracellular matrix (ECM) proteins (82). Fibrosis is brought on by cardiac inflammation, which triggers the production of growth factors and fibrotic cytokines, especially TGF-β1. This builds up fibrous collagen in the extracellular matrix, causing irreversible malfunction and eventually heart failure. Cardiac fibroblasts, the main cell type in injury-induced fibrosis, become activated and differentiate into myofibroblasts, which causes the matrix to become more rigid and the heart to function abnormally, ultimately leading to heart failure (84). According to the current histochemical results, the heart tissue of rats given TAA contained more collagen fibers. This result was supported by immunohistochemistry findings, which showed a rise in the TGF-β1 and NF-κB expression. Our results were in line with the results of Abdel-Rahman et al. (34), who reported that the NF-κB activated by TAA injection causes fibrosis advancement by activation of fibrogenic proteins in heart tissue. Also, rats given TAA exhibited marked collagen deposition in liver tissue with higher levels of TGF-β expression in mice (85) or NF-κB, p65, and collagen I expressions in rats (50). On the other hand, TAA and WP administration successfully modulated TAA-induced cardiac fibrosis as well as cardiac immunoreactivity of both NF-κB along with TGF-β marker expression and NF-κβ upregulation. According to Chen et al. (86), lactoferrin, a byproduct of WP, inhibits the proliferation, transformation, and collagen synthesis in cardiac fibrosis caused by NF-κB and TGF-β1.

A number of possible explanations for WP’s cardioprotective effects could come from either reducing inflammation or directly insulating cells and tissues from injury, as previously described (87). The first explanation could be due to the antioxidant effects of WPs, including reducing oxidative stress and providing antioxidant peptides that are important substrates for GSH production (88) in numerous studies of humans and animals (89). Recent research indicates that WP’s high content of sulfur-rich amino acids, like cysteine (90), as well as its antioxidant components (91), are linked to the formation of GSH. Numerous *in vitro* experiments using various cell lines administered with WP have also demonstrated the antioxidant properties of WP. Accordingly, O'Keeffe and Fitz (92) showed that hydrolyzed WP had a higher antioxidant capacity than undigested WP using the oxygen radical absorbency capacity assay. Additionally, they demonstrated favorable modulation of genes linked to antioxidant activity and enhanced GSH activity when hydrolyzed WPC was incubated with endothelial cells of human umbilical vein. Also, Ballatore et al. (93) showed that the effects of WP-derived peptides on oxidative stress have been investigated, exhibiting notable cytoprotective qualities, as evaluated using cell models and oxygen radical and hydroxyl radical absorbency capacity, as measured by ORAC assay. The second explanation could be linked to the bioactive compounds found in whey, such as immunoglobulins, *α*-lactalbumin, β-lactoglobulin, lactoferrin, hormones, growth factors, and lysozyme, which have multiple positive health effects, including preventing inflammation, infection, and cancer (94).

TAA toxicity typically results from oral, dermal, or pulmonary exposure. Intraperitoneal TAA toxicity can be a valuable experimental technique for producing a rapid or significant pattern of cardiac toxicity because oral TAA treatment often causes less extrahepatic toxicity. TAA, administered orally or intraperitoneally, is a safe and effective method for studying fibrosis in rodents with considerable variation in the dosage, timing, route, and animal (95).

In conclusion, it is commonly known that exposure to TAA causes an excess of reactive oxygen species, which can lead to cardiotoxicity. Also, WP’s antioxidant properties have the potential to alleviate oxidative stress and strengthen the antioxidant defense system, hence preventing oxidative heart injury. Notably, large amounts of whey protein are created as a waste product during the industrial processing of dairy products, and their improper disposal may have negative environmental effects. These peptides and their component parts are readily available, very inexpensive, and have excellent nutritional and biological value. Antioxidant and anti-inflammatory properties are advantageous as natural dietary additives and therapeutic components. At the molecular, histological, and biochemical levels, the results of this study clearly show that whey’s protective qualities can shield rats’ hearts against thioacetamide heart injury. This is because WP’s antioxidant properties help alleviate heart conditions brought on by TAA poisoning. Finding the heart’s antioxidant and ameliorative qualities is the only goal of the investigation. It will take further research for an accurate understanding of pathways of signaling and underlying mechanisms of action.

## Data Availability

The original contributions presented in the study are included in the article/Supplementary material, further inquiries can be directed to the corresponding author.
