# Supplementary material for: The cardioprotective effect of whey protein against thioacetamide-induced toxicity through its antioxidant, anti-inflammatory, and anti-apoptotic effects in male albino rats
Source: Front Vet Sci. 2025 May 19;12:1590722. doi: 10.3389/fvets.2025.1590722 (PMC12127417; doi:10.3389/fvets.2025.1590722)
Supplement: Supplementary file 1 [file Table_1.docx]

### Supplementary Table S1. ELISA Standard Curve Data

Mean absorbance values and standard deviations (±SD) for serial dilutions used to generate standard curves for each target analyte. Values are based on simulated triplicate readings.

| Analyte | Concentration (pg/mL) | Mean Absorbance (450 nm) | SD (±) |
| --- | --- | --- | --- |
| IL-1β | 0.0 | 0.05 | 0.003 |
| IL-1β | 15.6 | 0.12 | 0.006 |
| IL-1β | 62.5 | 0.3 | 0.015 |
| IL-1β | 250.0 | 0.78 | 0.039 |
| IL-1β | 1000.0 | 1.55 | 0.078 |
| TNF-α | 0.0 | 0.04 | 0.002 |
| TNF-α | 7.8 | 0.1 | 0.005 |
| TNF-α | 31.2 | 0.26 | 0.013 |
| TNF-α | 125.0 | 0.7 | 0.035 |
| TNF-α | 500.0 | 1.45 | 0.072 |
| Bcl-2 | 0.0 | 0.06 | 0.003 |
| Bcl-2 | 31.2 | 0.14 | 0.007 |
| Bcl-2 | 125.0 | 0.35 | 0.017 |
| Bcl-2 | 500.0 | 0.9 | 0.045 |
| Bcl-2 | 2000.0 | 1.7 | 0.085 |
| Bax | 0.0 | 0.05 | 0.003 |
| Bax | 62.5 | 0.13 | 0.007 |
| Bax | 250.0 | 0.33 | 0.016 |
| Bax | 1000.0 | 0.85 | 0.042 |
| Bax | 4000.0 | 1.65 | 0.082 |
