# Supplementary figures and images for: The cardioprotective effect of whey protein against thioacetamide-induced toxicity through its antioxidant, anti-inflammatory, and anti-apoptotic effects in male albino rats
Source: Front Vet Sci. 2025 May 19;12:1590722. doi: 10.3389/fvets.2025.1590722 (PMC12127417; doi:10.3389/fvets.2025.1590722)

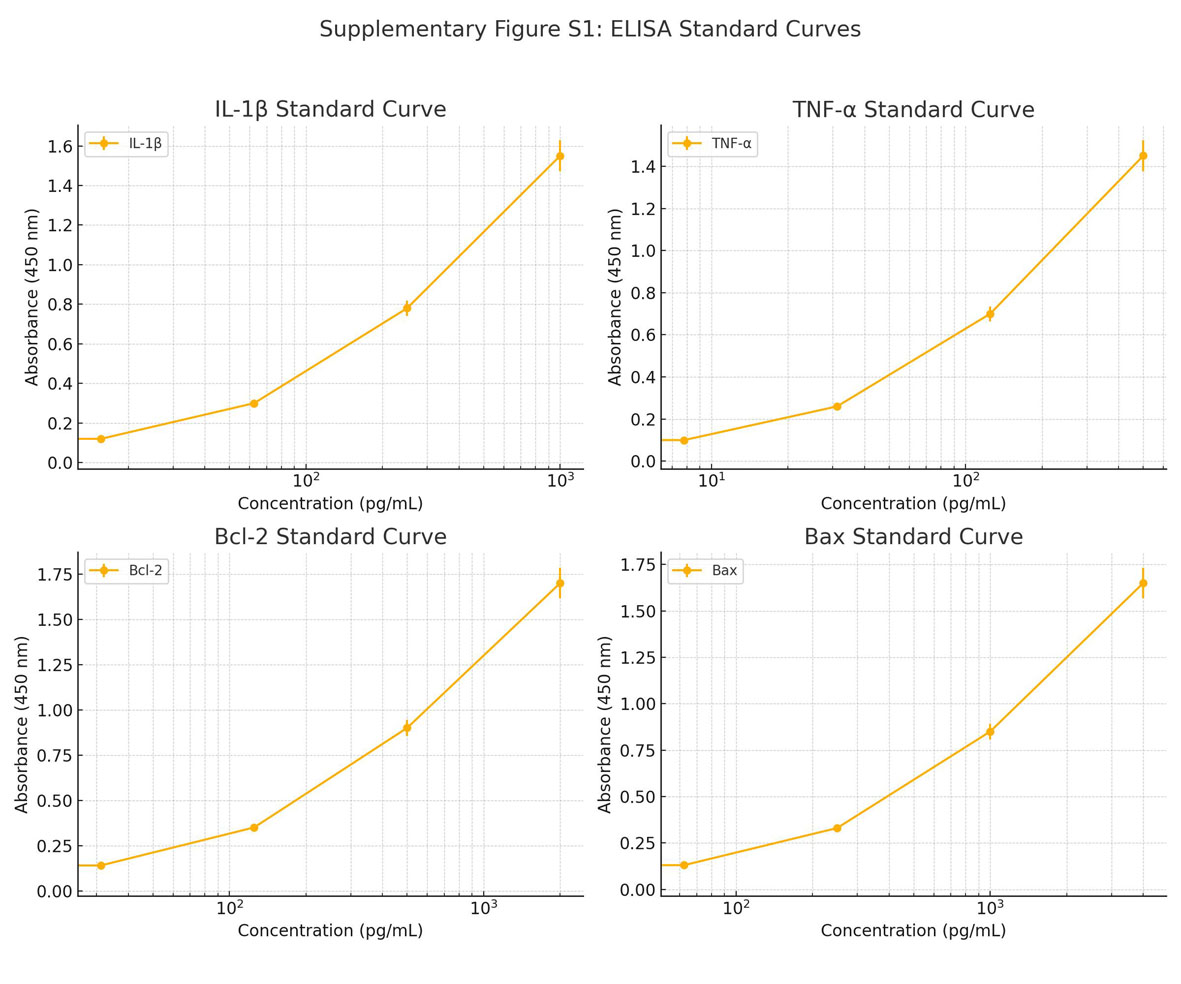

Supplement: Supplementary file 2 [file Image_1.jpeg]
